# A Novel Molecular Assay for Point-of-Care Diagnosis of Paracoccidioidomycosis from Clinical Samples Using Recombinase Polymerase Amplification Coupled with a Lateral Flow Assay

**DOI:** 10.3390/jof12070480

**Published:** 2026-07-01

**Authors:** Javier Mussin, Luis Corredor Sanguña, Florencia Dinorah Rojas, Diego Comerci, Gustavo Giusiano

**Affiliations:** 1 Consejo Nacional de Investigaciones Científicas y Técnicas (CONICET), Resistencia 3500, Argentina; luis.sanguna02@gmail.com (L.C.S.); florenciarojas@hotmail.com (F.D.R.); dcomerci@iib.unsam.edu.ar (D.C.); giusianogustavo@gmail.com (G.G.); 2 Instituto de Medicina Regional (IMR), Universidad Nacional del Nordeste (UNNE), Resistencia 3500, Argentina

**Keywords:** Paracoccidioidomycosis, recombinase polymerase amplification (RPA), lateral flow assay (LFA), point-of-care diagnosis, isothermal amplification, molecular diagnostics, endemic mycoses

## Abstract

Paracoccidioidomycosis (PCM) is a systemic mycosis endemic to Latin America. Its diagnosis is often delayed due to the limited availability of accessible and rapid molecular tools in endemic settings. In this study, we developed and conducted a preliminary evaluation of a recombinase polymerase amplification assay coupled with a lateral flow readout (RPA-LFA) for genus-level detection of *Paracoccidioides* spp. using reference strains and DNA extracted from clinical specimens. The assay targets the ITS1–5.8S–ITS2 ribosomal region using dual-labeled primers, which enable visual detection on LFA strips. Analytical specificity, analytical sensitivity, and clinical concordance were evaluated using reference strains, non-target microorganisms, and an exploratory set of clinical samples. No cross-reactivity was observed with *Coccidioides posadasii*, *Emmonsia crescens*, *Histoplasma capsulatum*, or *Leishmania braziliensis*. The preliminary analytical limit of detection (LOD) was 100 copies/µL. Visual results were obtained within 35 min, including 20 min of amplification at 39 °C and 15 min for LFA readout. In the clinical sample set analyzed, the assay showed complete concordance with direct microscopy. The RPA-LFA approach addresses several operational limitations of conventional molecular methods by combining isothermal amplification, a short turnaround time, visual interpretation, and low equipment requirements. This work provides a proof of concept for a point-of-care-oriented molecular approach for *Paracoccidioides* detection in clinical specimens. Further validation in larger and more diverse clinical cohorts is required to establish its diagnostic performance and potential implementation in endemic settings.

## 1. Introduction

Paracoccidioidomycosis (PCM) is a systemic fungal disease caused by thermally dimorphic species of the genus *Paracoccidioides*, mainly members of the *P. brasiliensis* complex and *P. lutzii* [1,2,3]. Endemic to Latin America, PCM is one of the most significant deep mycoses in the region due to its broad geographic distribution, chronic nature, and potential to cause severe illness when diagnosis and treatment are delayed [3,4,5,6]. Infection is acquired via the inhalation of environmental fungal propagules, which reach the lungs to initiate the primary pulmonary infection. PCM is not transmitted person to person or zoonotically, since the infection results from environmental exposure [2,3]. In many individuals, the fungus may remain latent in the lungs for years or even decades after the initial exposure before active disease develops [7]. The burden of PCM is particularly high in South America, where ecological, occupational, and socioeconomic conditions contribute to persistent exposure [4,8,9,10]. Most affected patients are adult males aged 30 to 60 years who are engaged in agricultural or other rural activities associated with frequent soil contact. PCM predominantly affects men, showing male-to-female ratios that can exceed 22:1, depending on the age group and geographic region. This epidemiological pattern has been associated with both occupational exposure and the protective effect of estrogens in women, which inhibit the transformation of the mycelial into the parasitic yeast phase. Clinically, PCM may present as chronic or acute/subacute forms with heterogeneous manifestations involving the lungs, mucous membranes, skin, lymph nodes, adrenal glands and other organs [2,3,8,11].

Despite its medical and social relevance, PCM is often overlooked and is still insufficiently prioritized in many public health agendas [2,9,10,12]. In endemic regions, patients frequently experience substantial diagnostic delays, particularly in rural or resource-constrained areas with limited access to specialized mycology laboratories [9,10,11,13]. These problematic delays are also due to the fact that PCM can mimic other infectious or non-infectious conditions. This hinders early clinical recognition and consequently delays the initiation of appropriate antifungal treatment [5,14]. Recognizing the growing importance of fungal diseases to global health, the World Health Organization (WHO) included *Paracoccidioides* in its 2022 list of fungal priority pathogens, underscoring the need to strengthen research, surveillance, and diagnostic capacity for this mycosis [15]. This scenario highlights a persistent gap: although PCM is a well-recognized endemic mycosis in Latin America, suitable diagnostic tools for timely and decentralized detection remain limited [2,9,10,15].

Laboratory diagnosis of PCM still relies mainly on conventional mycological methods, including direct microscopic examination and fungal culture. Direct microscopy can provide rapid evidence of infection when characteristic fungal elements are present. However, its sensitivity depends heavily on fungal burden, specimen quality, and operator expertise. False-negative results may therefore occur, especially in clinical specimens with low fungal load or heterogeneous distribution of fungal elements. Culture is highly specific but inherently slow and requires biosafety conditions and trained personnel, which may limit its routine use in laboratories serving endemic populations. Additionally, access to clinical samples for microbiological study is not always possible [2,3,13,16]. Serological methods are valuable for diagnosis and follow-up. Nevertheless, their performance may vary, influenced by immunological and procedural factors, as well as by antigenic differences among *Paracoccidioides* species and strains [17,18,19,20]. This variability may reduce sensitivity in some settings and complicate interpretation across regions where distinct phylogenetic lineages circulate [1,17,19,20,21].

Over the past two decades, molecular approaches have emerged as promising alternatives for PCM diagnosis [2,16,21]. Conventional PCR, nested PCR, and real-time PCR assays targeting ribosomal and other genomic regions have demonstrated high analytical sensitivity and specificity for detecting *Paracoccidioides* DNA [16,21,22,23]. These methods have also improved the molecular identification of the genus and expanded knowledge of its diversity and epidemiology. However, despite their strong analytical performance, PCR-based assays have not been widely incorporated into routine diagnostic workflows in many endemic areas [2,11,16,21]. Implementing them requires thermal cyclers, stable electricity, controlled laboratory conditions, contamination-control measures, and personnel trained in molecular biology techniques. Such requirements represent major barriers for peripheral or low-complexity healthcare facilities, precisely where earlier PCM diagnosis would be most valuable [2,9,10,12].

In this context, isothermal nucleic acid amplification technologies have gained increasing attention as a practical alternative to PCR for diagnosing infectious diseases in low-resource settings. Unlike PCR, these methods amplify nucleic acids at a constant temperature, thereby reducing instrument complexity and shortening assay turnaround time. Among them, recombinase polymerase amplification (RPA) is particularly attractive for point-of-care applications because it operates efficiently at relatively low temperatures, usually between 37 and 42 °C, and can generate detectable products within a short time [24,25]. When RPA is combined with a lateral flow assay (LFA), amplification products can be visualized on an immunochromatographic strip through an easy-to-interpret readout, avoiding the need for electrophoresis or complex optical equipment. This combination offers an important operational advantage for decentralized testing, as it couples the analytical power of nucleic acid amplification with the simplicity of a visually readable strip format. Although RPA-LFA platforms have already shown considerable promise for diagnosing a wide range of infectious agents, they have not yet been systematically developed for PCM diagnosis [24,25,26].

For a molecular assay intended for decentralized PCM detection, target selection is also critical. The ribosomal ITS1–5.8S–ITS2 has been extensively used for fungal identification and contains informative sequence variation that supports the detection and differentiation of *Paracoccidioides* from phylogenetically related fungi [21,22,23]. In addition, since this ribosomal region is present in multiple copies in the fungal genome, it may increase the likelihood of detection in clinical samples with low fungal biomass [21,22,23]. Previous studies have also demonstrated the utility of this region for the molecular detection of *Paracoccidioides*, including in tissue samples [22,23]. From a clinical perspective, genus-level detection may already be highly valuable in endemic settings, where the immediate priority is often the rapid confirmation of PCM in patients with compatible manifestations to support timely clinical management [2,3,12,21].

Based on these considerations, the aim of the present study was to develop and preliminarily evaluate a point-of-care-oriented RPA-LFA assay for genus-level detection of *Paracoccidioides* spp. using DNA extracted from clinical specimens. The platform was designed to provide rapid, genus-specific, and visually interpretable detection while reducing instrument requirements and operational complexity. This study provides a proof of concept for a potentially field-deployable molecular diagnostic workflow by integrating the speed of isothermal amplification with the portability of lateral flow detection. This system could contribute to earlier PCM diagnosis, improved access to laboratory confirmation, and strengthened epidemiological surveillance in endemic regions.

## 2. Materials and Methods

### 2.1. Microorganisms and Culture

The analytical performance of the assay was evaluated using reference strains and clinical samples. The fungal strains included *P. brasiliensis* S1 (IMR-Pb492), *P. brasiliensis* PS3 (Pb339), *P. lutzii* (Pb01), *Emmonsia crescens* (DMic 083307), and *Histoplasma capsulatum* (IMR-MF1190), obtained from the culture collection of the Department of Mycology, Instituto de Medicina Regional (IMR), Universidad Nacional del Nordeste (UNNE), Argentina. These organisms were selected because they included target *Paracoccidioides* isolates representing clinically relevant members of the genus, as well as phylogenetically related dimorphic fungi that may be considered in the differential diagnosis of PCM.

Strains of *Paracoccidioides*, *E. crescens*, and *H. capsulatum* were subcultured on Sabouraud dextrose agar and incubated at 37 °C for 21 days under conditions routinely used in the laboratory for maintenance of the yeast phase. In addition, genomic DNA of *Coccidioides posadasii* was kindly provided by Dr. Adriana Toranzo (Department of Mycology, INEI-ANLIS “Dr. Carlos G. Malbrán”, Argentina), and DNA of *Leishmania braziliensis* was kindly provided by Dr. Horacio Lucero (Department of Molecular Biology, IMR, UNNE). These non-target DNAs were included to assess potential cross-reactivity of the assay with clinically relevant microorganisms that may be considered in the differential diagnosis of PCM.

### 2.2. DNA Extraction

Total genomic DNA was extracted from all *Paracoccidioides* strains and non-target organisms using the DNeasy Blood & Tissue Kit (Qiagen, Hilden, Germany), following the manufacturer’s instructions. After extraction, DNA preparations were stored at −20 °C until use. Extracted DNA was used both for optimization of the amplification system and for assessment of analytical specificity and sensitivity.

For clinical specimens, total DNA was extracted individually using the same kit and according to the same manufacturer-recommended protocol in order to ensure consistency across the analytical and diagnostic evaluations. Purified DNA was subsequently used as a template for the RPA-LFA assay.

### 2.3. Primer Selection and 5′ Labelling for RPA–LFA

The ribosomal ITS1–5.8S–ITS2 region was selected as the molecular target for amplification. The primer pair used in this study was previously developed and analytically validated by our group in an earlier study [27] and was adopted here as the molecular basis for the RPA assay. For lateral flow detection, the same primer sequences were synthesised with 5′ terminal labels, incorporating biotin in the forward primer and digoxigenin in the reverse primer to generate a double-labelled amplification product compatible with immunochromatographic detection.

Primer compatibility with RPA chemistry was reassessed according to the recommendations provided by the manufacturer of the TwistAmp^®^ system (TwistDx Ltd., Cambridge, UK). In addition, primer specificity was re-evaluated in silico using the BLASTn algorithm available through NCBI GenBank in order to confirm genus-level specificity for *Paracoccidioides*. Primers were synthesised by LGC Biosearch Technologies (Petaluma, CA, USA). Their sequences and labels are shown in Table 1.

### 2.4. RPA Assay

RPA reactions were performed using the TwistAmp^®^ Basic Kit (TwistDx, Cambridge, UK) according to the manufacturer’s instructions. Each reaction had a final volume of 50 μL and consisted of 29.5 μL of rehydration buffer, 2.4 μL of 10 μM forward primer, 2.4 μL of 10 μM reverse primer, 12.2 μL of nuclease-free water, and 1 μL of DNA template. The reaction mixture was used to resuspend the lyophilised enzyme pellet provided in the kit, and amplification was initiated by adding 2.5 μL of 280 mM magnesium acetate.

Reactions were incubated at 39 °C for 20 min in a heating block. After amplification, products were immediately stored at −20 °C until analysis. Initial confirmation of successful amplification was performed by electrophoresis on 2% (*w*/*v*) agarose gel. The same amplification products were then subjected to lateral flow detection.

### 2.5. Lateral Flow Assay

The LFA strips were prepared and assembled as previously described [28,29], with minor modifications. Briefly, nitrocellulose membranes were striped with anti-digoxigenin antibody (0.5 mg mL^−1^) at the test line and biotin-BSA (1 mg mL^−1^) at the control line. Streptavidin-coated gold nanoparticles were diluted 1:5 in conjugation buffer consisting of 10 mM borate, pH 8.8, supplemented with 20% (*w*/*v*) sucrose. Then, 25 μL of this suspension was applied to the conjugate pad and allowed to air-dry at room temperature.

The nitrocellulose membrane, conjugate pad, sample pad, and absorbent pad were sequentially assembled onto a 5 mm adhesive backing card and cut into individual strips. The resulting format was designed to detect the double-labelled RPA amplicons generated by the biotin- and digoxigenin-modified primers.

For each test, 5 μL of RPA product was mixed with 75 μL of running buffer consisting of PBS (pH 7.4) supplemented with 1% (*w*/*v*) BSA and 0.05% (*v*/*v*) Tween 20 and then applied to the sample pad. The strip was subsequently placed vertically into a microtube containing 300 μL of running buffer, with the sample pad facing downwards to allow capillary migration. Results were recorded after 15 min of migration.

**Figure 1:**
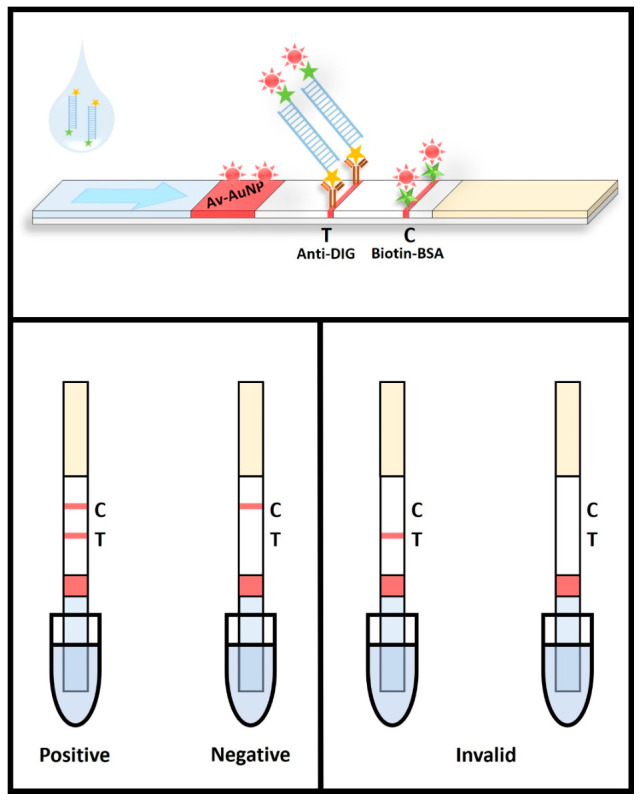
Schematic representation of the lateral flow assay (LFA) strip used to detect biotin- and digoxigenin-labelled amplicons generated by recombinase polymerase amplification (RPA). Illustration of the possible test outcomes. The double-labelled amplicon carries biotin at the 5′ end of the forward primer and digoxigenin at the 5′ end of the reverse primer. Streptavidin-coated gold nanoparticles (Av-AuNP) bind to the biotin moiety. The test line (T) consists of anti-digoxigenin antibody (anti-DIG), whereas the control line (C) consists of biotin–bovine serum albumin (biotin-BSA). A positive result shows both T and C lines; a negative result shows only the C line; and an invalid result shows no C line.

The assay outcome was interpreted visually and classified as positive when both test and control lines were visible, negative when only the control line was observed, and invalid when the control line failed to appear. A schematic representation of the detection principle and interpretation criteria is shown in Figure 1.

### 2.6. Analytical Specificity

The analytical specificity of the RPA-LFA assay for genus-level detection of *Paracoccidioides* was assessed using genomic DNA from the three target *Paracoccidioides* strains and from non-target organisms selected based on phylogenetic relatedness or clinical relevance in the differential diagnosis of PCM. These included *C. posadasii*, *E. crescens*, *H. capsulatum*, and *L. braziliensis*.

Each target and non-target DNA sample was tested in triplicate under the established RPA conditions, followed by visual detection on LFA strips. A no-template control was included in each assay run to monitor potential contamination. The assay was considered analytically specific when positive results were obtained exclusively for *Paracoccidioides* DNA, whereas all non-target samples and no-template controls yielded only the control line in all replicates.

### 2.7. Analytical Limit of Detection (LOD) Assessment

The analytical LOD assessment was performed using ten-fold serial dilutions of purified RPA amplicon DNA. For this purpose, genomic DNA from the reference strain Pb339 was first amplified by RPA, and the resulting amplicon was purified using the DNeasy Blood & Tissue Kit (Qiagen, Hilden, Germany) according to the manufacturer’s instructions. The purified DNA was then diluted 1:100 and quantified using a Qubit 4 fluorometer (Thermo Fisher Scientific, Waltham, MA, USA) together with a Qubit™ 1X dsDNA HS Assay Kit (Cat. No. Q33230; Invitrogen, Thermo Fisher Scientific, Eugene, OR, USA).

The DNA copy number per microlitre was calculated from the measured concentration using the following equation:

$$\mathrm{Copies}/\mu L=\frac{C\times (6.022\times 10^{23})}{N\times 660\times 10^{9}}$$

where

*C* = DNA concentration (ng/µL) as determined by Qubit

6.022 × 10^23^ = Avogadro’s number (molecules/mol)

*N* = length of the amplified fragment (bp)

660 = average molecular weight of a base pair (g/mol)

10^9^ = conversion factor from ng to g

Based on this calculation, ten-fold serial dilutions were prepared to obtain concentrations corresponding to 10,000, 1000, 100, 10, and 1 copy/μL. These dilutions were used as templates in the RPA-LFA assay to determine the lowest concentration consistently detectable under the established reaction conditions. Each dilution was tested in triplicate. Due to the limited number of replicates evaluated at each dilution level, this analysis was considered a preliminary analytical LOD assessment, defined as the lowest DNA concentration producing positive RPA-LFA results in all three technical replicates under the established experimental conditions. Therefore, the resulting value should be interpreted as an analytical estimate obtained under controlled laboratory conditions using purified DNA dilutions. It does not account for DNA extraction efficiency or matrix-associated inhibition.

### 2.8. Clinical Samples and Diagnostic Evaluation

To explore the diagnostic applicability of the assay, a total of eight clinical specimens from different patients were analysed. These included two lymph node aspirates, two sputum samples, two skin scrapings, and two skin biopsies. For each specimen type, one microscopy-positive and one microscopy-negative sample were included. Positive samples were obtained from patients with PCM confirmed by direct microscopic examination, whereas negative controls corresponded to samples from patients with alternative diagnoses.

This set of specimens was selected to represent different clinically relevant sample matrices commonly encountered in PCM diagnosis and to provide a preliminary assessment of assay performance across heterogeneous sample types. All samples were collected after written informed consent had been obtained and in accordance with institutional ethical requirements.

DNA extracted from each clinical specimen was used as a template in the RPA-LFA assay under the same reaction conditions described above. The test results were compared with the corresponding microscopy findings in order to assess preliminary concordance between the molecular assay and routine direct examination.

### 2.9. Ethical Considerations

The study was conducted in accordance with the Declaration of Helsinki and approved by the Ethics and Research Committee of the IMR, UNNE, Argentina (Renis CE000326). Informed consent was obtained from all subjects involved in the study prior to sample collection and analysis.

## 3. Results

### 3.1. RPA Amplification

**Figure 2:**
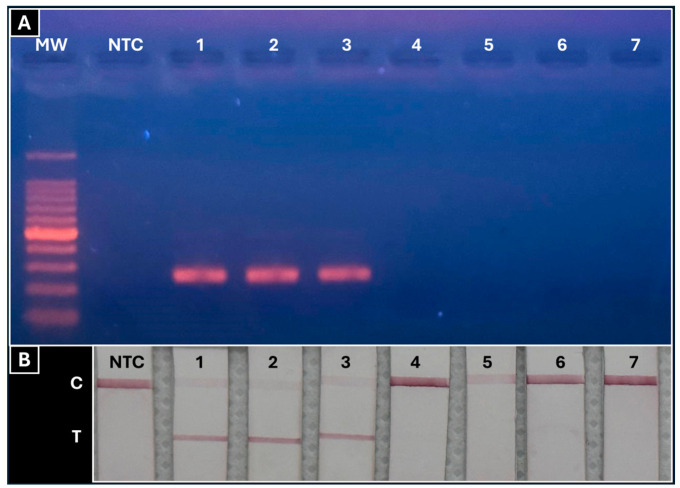
Agarose gel electrophoresis of RPA products (**A**) and lateral flow strip results (**B**) obtained for target and non-target microorganisms. MW, 100 bp DNA ladder. The expected amplicon size is 242 bp. NTC: no-template control. Lane/strip 1: *P. brasiliensis* S1; lane/strip 2: *P. brasiliensis* PS3; lane/strip 3: *P. lutzii*; lane/strip 4: *C. posadasii*; lane/strip 5: *H. capsulatum*; lane/strip 6: *E. crescens*; lane/strip 7: *L. braziliensis*. Each sample was tested in triplicate; the qualitative results were concordant across all three replicates, and representative strips are shown.

RPA amplification was first evaluated using genomic DNA from the *Paracoccidioides* strains included in the study. Reactions performed with *P. brasiliensis* S1 (IMR-Pb492), *P. brasiliensis* PS3 (Pb339), and *P. lutzii* (Pb01) produced amplification products of the expected size (approximately 242 bp), as observed by agarose gel electrophoresis (Figure 2A). The presence of a single visible band close to the expected molecular size confirmed successful amplification of the selected ITS1–5.8S–ITS2 target region under the established RPA conditions.

No amplification was observed in the no-template control or in reactions containing DNA from non-target microorganisms. These results indicated that the selected reaction conditions for the assay were suitable for amplifying *Paracoccidioides* DNA and did not generate detectable nonspecific products under the tested experimental conditions. The consistent amplification observed for the three *Paracoccidioides* strains also supports the ability of the primer pair to detect different members of the genus, including representatives of the *P. brasiliensis* complex and *P. lutzii*.

The amplification products obtained by RPA were subsequently used for LFA detection in order to evaluate whether the double-labelled amplicons generated by the biotin- and digoxigenin-modified primers could be visually detected on the immunochromatographic strips.

### 3.2. Analytical Specificity of the RPA–LFA Assay

The analytical specificity of the RPA-LFA assay was assessed using genomic DNA from target and non-target microorganisms. A positive visual result, defined as the simultaneous appearance of both the test and control lines, was observed exclusively in reactions containing DNA from *Paracoccidioides* spp. (Figure 2B). All target *Paracoccidioides* strains yielded positive RPA-LFA results in all replicates.

In contrast, DNA from the non-target microorganisms tested, including *C. posadasii*, *E. crescens*, *H. capsulatum*, and *L. braziliensis*, produced negative results in all replicates, characterized by the presence of only the control line. No-template controls also remained negative in all assay runs. No invalid results were observed, as the control line appeared in all strips tested. These findings indicate that no cross-reactivity was observed with the non-target microorganisms included in the analytical specificity panel under the conditions evaluated.

### 3.3. Preliminary Analytical LOD Assessment

The analytical sensitivity of the RPA-LFA assay was preliminarily evaluated using ten-fold serial dilutions of purified DNA corresponding to 10^4^, 10^3^, 10^2^, 10, and 1 copy/µL. Each dilution was tested in triplicate to determine the lowest concentration that could be consistently detected under the experimental conditions evaluated.

**Figure 3:**
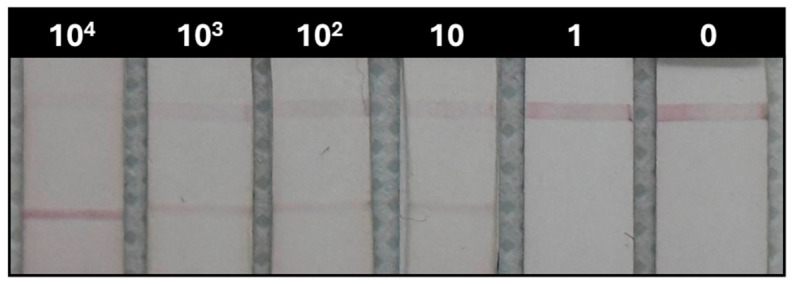
Results of the RPA–LFA using serial dilutions of purified DNA corresponding to 10^4^; 10^3^; 10^2^; 10; 1; and 0 copies per microliter.

Positive LFA results were obtained in all replicates containing 10^4^, 10^3^, and 100 copies/µL (Figure 3; Table 2). At these concentrations, the strips showed both the test and control lines, indicating successful amplification and lateral flow detection of the double-labelled amplicons. Under the experimental conditions evaluated, the lowest concentration consistently detected in all three replicates was 100 copies/µL.

At lower DNA concentrations, detection became inconsistent. At 10 copies/µL, only one of the three replicates produced a detectable positive result, corresponding to a detection frequency of 33%. At 1 copy/µL, no positive results were observed. The negative control also remained negative in all replicates. These results indicate that although occasional amplification may occur below 100 copies/µL, reliable visual detection by RPA-LFA was achieved only at concentrations equal to or above 100 copies/µL.

Based on these findings, the preliminary analytical LOD of the RPA-LFA assay was defined as 100 copies/µL. This value should be interpreted as an analytical estimate obtained under controlled laboratory conditions using purified DNA dilutions.

### 3.4. Detection of Paracoccidioides DNA in Clinical Samples

The preliminary diagnostic applicability of the RPA-LFA assay was evaluated using eight clinical specimens obtained from different patients. The sample panel included two lymph node aspirates, two sputum samples, two skin scrapings, and two skin biopsies. For each specimen type, one microscopy-positive and one microscopy-negative sample were included.

All microscopy-positive samples yielded positive RPA-LFA results, as evidenced by the presence of both test and control lines on the strips. Conversely, all microscopy-negative samples yielded negative RPA-LFA results, with visualization of the control line only. No discordant results were observed between RPA-LFA and direct microscopy within this preliminary sample set, and no invalid tests occurred.

Overall, the RPA-LFA assay showed complete concordance with direct microscopic examination across all specimen types evaluated (Table 3). The overall concordance with direct microscopy was 8/8 (100%) in this exploratory evaluation. These findings suggest that the assay can detect *Paracoccidioides* DNA across heterogeneous clinical matrices commonly encountered in PCM diagnosis. However, given the limited number of clinical specimens, these results should be interpreted as preliminary evidence of clinical feasibility rather than as definitive estimates of diagnostic sensitivity, specificity, or clinical performance.

**Figure 4:**
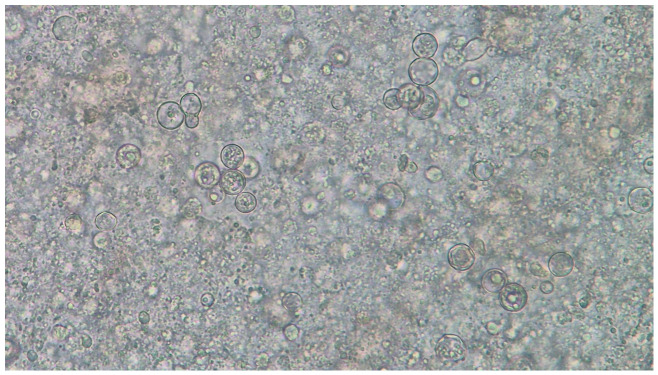
Direct-microscopy view (400×) of the lymph node aspirate included in the clinical evaluation summarized in Table 3, showing the typical multi-budding yeast cells of *Paracoccidioides*. This same specimen yielded a positive result by RPA-LFA.

Figure 4 shows the direct microscopic examination of the lymph node aspirate included in Table 3, revealing multi-budding yeast cells consistent with *Paracoccidioides* spp. This illustrates the concordance between the microscopy of the positive clinical samples included in this study and the positive RPA-LFA test result obtained in the preliminary clinical evaluation.

## 4. Discussion

This study describes the development and preliminary evaluation of an RPA assay coupled with an LFA readout for the rapid detection of *Paracoccidioides* spp. DNA from clinical samples. The assay combines the analytical advantages of nucleic acid amplification with the operational simplicity of visual lateral flow detection. Under the evaluated conditions, the RPA-LFA demonstrated genus-level detection of *Paracoccidioides* spp., a preliminary analytical LOD of 100 copies/µL, and complete concordance with direct microscopy in the exploratory clinical sample set analyzed. These findings support that using an isothermal amplification strategy coupled with a portable visual readout is a feasible and practical molecular approach for PCM diagnosis.

Despite advances in diagnostic methodologies, the diagnosis of PCM remains challenging in endemic regions, particularly in areas with restricted access to specialized mycology facilities. In routine practice, conventional diagnostic methods such as direct microscopic examination and fungal culture remain the most widely available diagnostic tools. Direct microscopy provides rapid evidence of infection when characteristic *Paracoccidioides* yeast cells are identified, although its diagnostic yield is strongly influenced by specimen quality, fungal load, and operator expertise. Fungal culture offers high specificity but is constrained by prolonged incubation times and the requirement for appropriate laboratory infrastructure and biosafety conditions, factors that may significantly delay confirmation of the diagnosis [2,3,16]. Serological assays constitute an important complementary approach and, in some cases, may represent the only accessible diagnostic resource. Nevertheless, their performance may be affected by multiple variables, including methodological, host immune status, and antigenic variability among circulating *Paracoccidioides* lineages [17,18,19,20]. Collectively, these limitations highlight the persistent difficulties associated with timely PCM diagnosis, particularly in resource-limited settings where early therapeutic intervention is critical.

Molecular approaches have considerably expanded the capacity for sensitive and specific detection of *Paracoccidioides* DNA. PCR-based assays targeting ribosomal or other genomic regions have shown high analytical performance [16,21,22,23]. However, despite their diagnostic potential, the implementation of these methods in routine practice remains limited across endemic settings. Their dependence on specialized professionals and laboratory infrastructure capable of supporting molecular workflows restricts their applicability in decentralized or low-resource environments [2,16,21]. In this context, the development of simplified and accessible molecular platforms represents a relevant strategy to facilitate broader access to PCM diagnosis and to support earlier detection in endemic regions.

In this context, RPA offers several operational advantages over conventional PCR, which are particularly relevant for decentralized diagnosis. Unlike PCR, RPA does not require repeated thermal cycling, as the reaction occurs under isothermal conditions, usually between 37 and 42 °C. This allows amplification to be performed using simple heating devices rather than a thermal cycler, reducing equipment requirements and improving portability. In addition, RPA is characterized by short reaction times, often generating detectable products within 10–30 min, which is compatible with rapid diagnostic workflows [24,25]. Another practical advantage is that RPA relies on a conventional primer-pair design, which may simplify assay development compared with more complex isothermal strategies such as loop-mediated isothermal amplification (LAMP). RPA is also highly compatible with lateral flow detection, enabling the conversion of amplification products into a visual signal that can be interpreted without electrophoresis or fluorescence-based instruments [24,26]. Furthermore, RPA has been reported to tolerate partially purified or minimally processed samples better than some PCR-based approaches, which is particularly important for future integration with simplified DNA extraction or crude lysis protocols [24,25]. Taken together, these features make RPA especially suitable for the development of rapid, portable, and low-complexity molecular assays intended for use outside of specialized laboratories.

The RPA-LFA platform developed in this study addresses certain practical limitations. The total assay time was approximately 35 min, including 20 min of amplification at 39 °C and 15 min for lateral flow migration and visual interpretation. The assay is intended as a point-of-care-oriented diagnostic tool. Although the current turnaround time of approximately 35 min is appropriate for near-patient diagnostic scenarios, future iterations should focus on shortening the lateral flow readout and integrating rapid lysis-based sample preparation to align the workflow with international standards for point-of-care and rapid diagnostic tests, thereby increasing its acceptance among healthcare professionals. Reducing the assay duration would reinforce its competitiveness relative to existing rapid tests and maximize its impact in real clinical and public health settings. Additionally, using an LFA readout simplifies result interpretation by converting the molecular amplification event into a visible signal. A positive result is defined by the simultaneous appearance of the test and control lines, while a negative result shows only the control line. This format reduces dependence on specialized equipment and makes the assay potentially suitable for laboratories with limited infrastructure.

The analytical specificity observed in this work is crucial. The assay detected DNA from *P. brasiliensis* S1, *P. brasiliensis* PS3, and *P. lutzii*, while no cross-reactivity was observed with *C. posadasii*, *E. crescens*, *H. capsulatum*, or *L. braziliensis*. The inclusion of these non-target microorganisms is important because dimorphic fungi and other clinically relevant microorganisms, such as *Leishmania*, are frequently considered in the differential diagnosis of PCM, particularly in endemic regions where the clinical manifestations overlap [2,3]. These findings support the preliminary genus-level specificity of the assay, although broader specificity panels including additional fungal and non-fungal microorganisms are required for a more comprehensive assessment of specificity.

Selecting the ITS1–5.8S–ITS2 ribosomal region as the amplification target also appears to be a key factor in assay performance. This genomic region has been extensively employed for fungal identification and has been demonstrated to be useful for the molecular detection of *Paracoccidioides* [16,17,18]. Due to its multicopy nature within the fungal genome, targeting the ITS region may enhance analytical sensitivity, particularly in clinical specimens containing a low fungal burden. This characteristic is especially relevant in samples in which direct microscopic examination is negative or inconclusive. Furthermore, the sequence variability within the ITS region enables discrimination between *Paracoccidioides* and phylogenetically related fungi, thereby supporting the level of specificity required for a clinically applicable diagnostic method.

The preliminary analytical sensitivity assessment revealed that, under the evaluated experimental conditions, the assay consistently detected 100 copies/µL. The detection pattern observed in the dilution assays was consistent with this threshold for all samples. All replicates were positive at 10,000, 1000, and 100 copies/µL, whereas detection became inconsistent at 10 copies/µL and was absent at 1 copy/µL. Since this value was determined using triplicate testing of purified DNA dilutions, it should be interpreted as a preliminary analytical LOD rather than as a direct measure of clinical sensitivity.

The evaluation of clinical specimens provides initial evidence that the assay can detect *Paracoccidioides* DNA in different types of samples. In this study, RPA-LFA results were concordant with those from microscopy for lymph node aspirates, sputum, skin scrapings, and skin biopsies. The inclusion of these sample types is relevant because PCM can affect several anatomical sites, and the diagnostic material available may vary according to the clinical presentation. The ability to obtain positive results from heterogeneous clinical matrices suggests that the assay may be adaptable to different diagnostic contexts. However, as only eight clinical specimens were included, these findings should be considered proof-of-concept evidence rather than definitive estimates of diagnostic sensitivity and specificity.

A significant contribution of this work is the integration of RPA with an LFA format for direct clinical testing. Our group previously developed a carbon nanoparticle-based PCR-LFA system for detecting specific double-tagged DNA amplicons of *Paracoccidioides* spp. [28]. The present approach builds on the lateral flow detection concept by replacing PCR amplification with RPA. In this sense, the current assay represents a step towards a more field-deployable molecular diagnostic workflow. The use of dual-labelled primers, with biotin and digoxigenin at the 5′ ends, allowed the generation of amplicons suitable for immunochromatographic capture and visual detection while preserving the simplicity of the strip-based format.

From a clinical perspective, a genus-level assay could be a highly useful initial diagnostic tool in endemic areas. While species-level identification is valuable for epidemiological studies and for understanding the distribution of Paracoccidioides lineages, the clinical priority is often to confirm the presence of Paracoccidioides DNA in patients with compatible manifestations. In this context, rapid genus-level detection could support early therapeutic decisions and reduce diagnostic uncertainty. This is particularly important since PCM may present with a wide spectrum of pulmonary, cutaneous, mucocutaneous, and lymphatic manifestations that overlap with those of other infectious and non-infectious diseases [2,3,12].

The potential public health value of this type of assay should also be emphasized. PCM remains underdiagnosed in endemic areas, and epidemiological surveillance is often limited by restricted access to reliable confirmatory diagnostic methods [2,9,10,15]. In this context, a rapid, portable, and visually interpretable molecular assay could contribute to the decentralization of diagnostic capacity, supporting earlier case detection and strengthening surveillance activities in resource-limited settings. Importantly, these features are consistent with the ASSURED framework proposed by the WHO Special Programme for Research and Training in Tropical Diseases (TDR) for point-of-care diagnostics intended for use in low-resource environments, particularly regarding affordability, user-friendliness, rapidity, robustness, minimal equipment requirements, and deliverability to end-user settings [30]. Furthermore, this approach aligns with priorities highlighted by the WHO for fungal priority pathogens, which include the need to improve diagnostic capacity, surveillance, and access to appropriate tools for clinically relevant fungal diseases [15]. Although further validation is still required, the present assay provides a methodological basis that could contribute to broader strategies aimed at improving the diagnosis and surveillance of endemic mycoses.

Despite these promising findings, several limitations of the present study should be acknowledged. First, the number of clinical samples evaluated was limited, which prevents robust estimation of diagnostic sensitivity, specificity, predictive values, and performance across different disease forms. Although a high concordance with direct microscopy was observed, these findings should not be interpreted as definitive evidence of clinical accuracy. Larger studies including well-characterized cohorts and different clinical presentations are needed to establish the true diagnostic performance of the assay across samples with different fungal burdens.

Second, the clinical evaluation was based on comparison with direct microscopy. Although microscopy is a conventional and widely used diagnostic method, it has recognized limitations as a reference standard. Future studies should compare RPA-LFA results with culture, serology, histopathology, conventional PCR, and real-time PCR whenever available. Such comparative analyses would provide a more comprehensive assessment of sensitivity and specificity, particularly in cases with negative microscopy but strong clinical suspicion of PCM. These studies will also be important to determine whether the RPA-LFA is capable of identifying cases that may be missed by direct examination.

Third, although the analytical specificity panel included clinically relevant non-target microorganisms, its scope remained limited. Additional validation should include a broader panel of fungi and other pathogens that may be present in clinical specimens or considered in the differential diagnosis of PCM. Expanding the specificity panel would further strengthen confidence in the assay’s performance under different diagnostic scenarios.

Fourth, the assay currently relies on purified DNA obtained using a commercial extraction kit. Although appropriate for analytical validation and controlled laboratory testing, this approach does not fully represent a simplified point-of-care workflow. For implementation in peripheral laboratories or field settings, future studies should evaluate rapid and low-cost DNA extraction methods compatible with RPA-LFA. The development of simplified sample preparation strategies will be essential to reduce processing time, cost, and equipment requirements. In particular, thermal, chemical, or minimal-processing lysis protocols could improve the feasibility of assay deployment in resource-limited settings.

Finally, while visual interpretation of LFA results is practical and user-friendly, faint test or control lines may introduce observer-dependent variability, particularly near the analytical limit of detection or when intensity is reduced. In the present study, the preliminary analytical LOD was conservatively established based on consistent detection across triplicate testing of purified DNA dilutions. In addition, although reduced control-line intensity was observed in some positive strips, the control line remained visible in all positive strips evaluated, and no invalid results were observed. Therefore, under the conditions tested, these visual features did not affect the result interpretation. Nevertheless, further optimization is warranted to improve control-line robustness. Future evaluations should assess key strip and assay parameters, such as the amount of RPA product applied to the strip, pre-migration dilution of the amplified product, streptavidin–gold conjugate concentration, biotin-BSA density at the control line, primer concentration, and running buffer conditions. In parallel, standardized visual scoring, independent readers, portable digital readers, or smartphone-assisted interpretation strategies could facilitate result documentation, semi-quantitative analysis, remote reporting, and reproducible interpretation in surveillance programs or field-based studies [28].

For a diagnostic platform intended for decentralized use, reagent stability, storage temperature, and ease of handling represent important considerations. RPA reagents and LFA strips should be evaluated under conditions that resemble those encountered in endemic regions, including fluctuating ambient temperatures and limited cold-chain availability. Such operational assessments would provide valuable information regarding the robustness and practical applicability of the assay outside reference laboratory settings.

Taken together, the findings of the present study support the potential utility of an RPA-LFA platform for detecting *Paracoccidioides* spp. in clinical specimens. The assay combines rapid isothermal amplification, genus-level specificity, visual readout, and compatibility with low-complexity equipment. These characteristics are particularly relevant for decentralized or resource-limited diagnostic settings. While additional validation is required before routine implementation, the present work provides a proof of concept for a feasible molecular approach for PCM diagnosis and establishes a foundation for future multicenter, prospective, and field-based evaluations.

## 5. Conclusions

The RPA-LFA assay developed in this study represents a promising, rapid, genus-specific, and visually interpretable molecular approach for the detection of *Paracoccidioides* spp. DNA extracted from clinical specimens. By combining isothermal amplification with lateral flow detection, the assay reduces dependence on complex laboratory equipment and offers a practical alternative to conventional PCR-based approaches, particularly in scenarios with limited molecular infrastructure. Under the evaluated conditions, the platform showed genus-level specificity, a preliminary analytical LOD of 100 copies/µL, and complete concordance with direct microscopy in the exploratory clinical sample set analyzed.

The operational characteristics of the assay, including its short turnaround time, low-temperature amplification requirements, and simple visual readout, support its potential applicability as a point-of-care or near-patient diagnostic strategy for PCM. These features are particularly relevant in endemic regions where access to specialized mycology laboratories is limited, and diagnostic delays remain a major challenge. In this context, the RPA-LFA approach could contribute to earlier laboratory confirmation, more timely clinical decision-making, and improved epidemiological surveillance of PCM. However, its performance must be confirmed in larger validation studies.

Nevertheless, the present study should be interpreted as a proof of concept rather than a definitive clinical validation. Further validation involving larger and more diverse clinical cohorts is required to establish robust estimates of diagnostic sensitivity, specificity, and predictive values. Future studies should also evaluate simplified DNA extraction protocols, expanded specificity panels, inter-operator reproducibility, incorporation of internal amplification controls, and field-based performance under real implementation conditions. Such advances will be essential to determine the practical scalability and clinical applicability of this platform for decentralized PCM diagnosis.

## Figures and Tables

**Table 1:** Sequences of the primers for RPA-LFA.

5′-Label	Sequence (5′-3′)	Type	Size (bp)
Biotin	CGTGCCCGCCGGGGACACCGTTGAACTTCTGGTTC	Forward	242
Digoxigenin	CGCTTGAGGGTTGAAATGACGCTCGGACAGGCATG	Reverse	

**Table 2:** Detection frequency of the RPA-LFA assay using ten-fold serial dilutions of purified DNA.

DNA Concentration (Copies/µL)	No. of Positive Replicates/Total	Detection Frequency (%)
10,000	3/3	100
1000	3/3	100
100	3/3	100
10	1/3	33
1	0/3	0
0 (Negative control)	0/3	0

**Table 3:** Concordance between RPA-LFA and direct microscopy according to clinical specimen type.

Clinical Specimen Type	No. of Samples	Microscopy-Positive/RPA-LFA Positive	Microscopy-Negative/RPA-LFA Negative	Concordance
Lymph node aspirate	2	1/1	1/1	2/2
Sputum	2	1/1	1/1	2/2
Skin scraping	2	1/1	1/1	2/2
Skin biopsy	2	1/1	1/1	2/2
**Total**	**8**	**4/4**	**4/4**	**8/8**
**Overall concordance with direct microscopy: 8/8, 100%.**				

## Data Availability

All data generated or analyzed during this study are included within the article. Additional information is available from the corresponding author upon reasonable request.
